# In Vitro Effect of Δ9-Tetrahydrocannabinol and Cannabidiol on Cancer-Associated Fibroblasts Isolated from Lung Cancer

**DOI:** 10.3390/ijms23126766

**Published:** 2022-06-17

**Authors:** Lara Milián, Irene Monleón-Guinot, María Sancho-Tello, José Marcelo Galbis, Antonio Cremades, María Almenar-Ordaz, Josep Peñaroja-Martinez, Rosa Farras, José Javier Martín de Llano, Carmen Carda, Manuel Mata

**Affiliations:** 1 Department of Pathology, Faculty of Medicine and Dentistry, Universitat de València, 46010 Valencia, Spain; lara.milian@uv.es (L.M.); irene.monleon@gmail.com (I.M.-G.); maria.sancho-tello@uv.es (M.S.-T.); malor4@alumni.uv.es (M.A.-O.); jopemar8@alumni.uv.es (J.P.-M.); mardella@uv.es (J.J.M.d.L.); carmen.carda@uv.es (C.C.); 2 INCLIVA Biomedical Research Institute, 46010 Valencia, Spain; 3 Hospital de la Ribera, 46600 Alzira, Spain; josegalbiscar@icloud.com (J.M.G.); antonio.cremades@uv.es (A.C.); 4 Príncipe Felipe Research Center Foundation (CIPF), 46012 Valencia, Spain; rfarras@cipf.es; 5 Biomedical Research Networking Center on Bioengineering, Biomaterials and Nanomedicina (CIBER-BBN), 28029 Madrid, Spain; 6 Biomedical Research Networking Center of Respiratory Diseases (CIBERES), 28029 Madrid, Spain

**Keywords:** lung cancer, EMT, TME, cannabinoid agonists, THC, CBD, CAFs, NFs

## Abstract

There is evidence that demonstrates the effect of cannabinoid agonists inhibiting relevant aspects in lung cancer, such as proliferation or epithelial-to-mesenchymal transition (EMT). Most of these studies are based on evidence observed in in vitro models developed on cancer cell lines. These studies do not consider the complexity of the tumor microenvironment (TME). One of the main components of the TME is cancer-associated fibroblasts (CAFs), cells that are relevant in the control of proliferation and metastasis in lung cancer. In this work, we evaluated the direct effects of two cannabinoid agonists, tetrahydrocannabinol (THC) and cannabidiol (CBD), used alone or in combination, on CAFs and non-tumor normal fibroblasts (NFs) isolated from adenocarcinoma or from healthy lung tissue from the same patients. We observed that these compounds decrease cell density in vitro and inhibit the increase in the relative expression of type 1 collagen (COL1A1) and fibroblast-specific protein 1 (FSP1) induced by transforming growth factor beta (TGFβ). On the other hand, we studied whether THC and CBD could modulate the interactions between CAFs or NFs and cancer cells. We conditioned the culture medium with stromal cells treated or not with THC and/or CBD and cultured A549 cells with them. We found that culture media conditioned with CAFs or NFs increased cell density, induced morphological changes consistent with EMT, inhibited cadherin-1 (CDH1) gene expression, and induced an increase in the relative expression of cadherin-2 (CDH2) and vimentin (VIM) genes in A549 cells. These changes were inhibited or decreased by THC and CBD administered alone or in combination. In another series of experiments, we conditioned culture media with A549 cells treated or not with THC and/or CBD, in the presence or absence of TGFβ. We observed that culture media conditioned with A549 in the presence of TGFβ induced an increase in the expression of COL1A1 and VIM, both in CAFs and in non-tumor NFs. Both THC and CBD ameliorated these effects. In summary, the results presented here reinforce the usefulness of cannabinoid agonists for the treatment of some relevant aspects of lung cancer pathology, and demonstrate in a novel way their possible effects on CAFs as a result of their relationship with cancer cells. Likewise, the results reinforce the usefulness of the combined use of THC and CBD, which has important advantages in relation to the possibility of using lower doses, thus minimizing the psychoactive effects of THC.

## 1. Introduction

Lung cancer is the leading cause of cancer death worldwide, which has led to several important public health problems [1,2]. Among non-small cell lung cancer (NSCLC) variants, lung adenocarcinoma is the most common histological subtype, the morbidity of which has been increasing year after year [2,3]. Chemotherapy is the main treatment for this and other types of cancer [4]; however, sometimes treatment fails due to drug resistance, leading to recurrence, malignant tumor progression, metastasis, and sometimes death [3,4,5,6].

There are multiple factors that determine not only the capability of lung cancer to generate resistance, but also its great ability to escape from the immune system and to invade, which allows it to nest in different organs and generate metastases [4,5]. In this sense, it is necessary to consider not only the genetic and epigenetic factors that affect specific clones of tumor cells, but also the complexity of the tumor microenvironment (TME), which includes malignant cells together with cells of the immune system, blood vessels, components of the extracellular matrix, mesenchymal cells, and cancer-associated fibroblasts (CAFs), which together determine the tumor stroma [5,6,7]. Interactions between cancer cells and the surrounding stroma support tumor establishment by recruiting and altering normal cells to participate in the disease [8].

CAFs are key elements in the control of the tumor stroma. They can express and secrete signaling proteins, including mitogenic epithelial growth factors, with the ability to promote the growth of primary tumors and establish a niche for cancer metastasis [8,9]. CAFs also contribute to the production of functional growth cytokines and exosomes, which interact with tumor cells and can suppress the immune response, creating an environment that is prone to tumor survival [9,10,11,12,13]. In addition, CAFs modify the tumor stroma and induce the epithelial-to-mesenchymal transition (EMT) through the secretion of different factors, including transforming growth factor beta (TGFβ) [8,9]. These factors, together with others such as physical forces, determine the great capability of lung cancer to generate resistance against antitumor agents, which has led different research groups, including ours, to search for new pharmacological targets complementary to those already existing [10,11].

Cannabinoids encompass a large group of organic molecules that are physiologically produced in humans, can be synthesized in laboratories, or can also be extracted from the Cannabis sativa plant, since they are its main active ingredient [12]. These molecules and their derivatives are generating great interest due to their apparent potential to interfere with tumor spread, growth, migration, and metastasis, in addition to their action as anti-inflammatory agents that suppress the antitumor immune response [14,15,16,17]. Cannabinoids interact with two specific cellular receptors, which belong to the family of transmembrane proteins called surface G protein-coupled receptors. The type 1 receptor (CB1) is expressed on all the cells of the central nervous system (CNS), and the type 2 receptor (CB2) is found primarily on hematopoietic and immune system cells. Both CB1 and CB2 seem to be expressed in many types of cancer, including lung cancer [11,14,15,16,17], supporting the hypothesis that cannabinoids might interfere with tumors by acting on CB1 and CB2 receptors.

The best known cannabinoids are Δ9 tetrahydrocannabinol (THC) and cannabidiol (CBD). THC has psychoactive effects due to its interaction with the CB1 receptor, while its immunomodulatory properties come from its interaction with the CB2 receptor [11]. The first evidence of the ability of THC to reduce the growth of lung adenocarcinoma, both in vitro and in vivo, was reported by Munson et al. in 1975 [18], and subsequently several preclinical studies have supported this result, suggesting that this compound could have some interesting properties in terms of the inhibition of tumor cell proliferation [19]. However, its use is still limited due to its psychoactive side effects [17].

By contrast, CBD is completely non-psychoactive. Unlike THC, the affinity of CBD for the CB1 and CB2 receptors is considered to be relatively low, although both CB1 and CB2 could still be the targets of CBD in certain cancer cells and in cells that infiltrate the TME [11,12,13,14,15,16,17]. CBD has been shown to have antiproliferative and proapoptotic functions in various human cancer cell lines and in mouse tumor models. In comparison, CBD generally has milder effects on normal cells in the same tissue/organ. In addition, CBD can also inhibit tumor cell migration as well as invasion and neovascularization, suggesting that CBD not only acts on tumor cells, but also affects the TME by modulating infiltrating mesenchymal and immune cells, inhibiting EMT and inducing the reversion to a non-invasive phenotype [11,16,17,18,19,20,21].

Previously, we found evidence that the combined use of THC and CBD inhibited proliferation and EMT in different lung cancer cell lines, including A549, H460 and H1700 [21]. Furthermore, we demonstrated the additive effect of the THC + CBD combination on the inhibition of proliferation and EMT in cancer cells. Although these data are consistent with previously reported data, it should be noted that they are based on studies carried out with cell lines, which therefore do not adequately represent the full complexity of the TME. To our knowledge, there are no previous studies on the effect of these compounds on CAFs or the interaction between CAFs and cancer cells.

Our objective in this work is, on the one hand, to study the effect of THC and CBD on CAFs isolated from the tumors of patients diagnosed with lung adenocarcinoma. On the other hand, we intend to evaluate if these drugs can modulate the interactions between cancer cells and CAFs. To carry out this study, we developed a simple system that consists of culturing CAFs from lung adenocarcinomas with A549 cell-conditioned culture medium, treated with TGFβ in the presence or absence of THC and CBD, alone or in combination. The results presented here indicate a beneficial effect of these cannabinoids in the modulation of some aspects of the tumor microenvironment.

## 2. Materials and Methods

### 2.1. Experimental Design

Our first objective in this work was to study the effect of THC and CBD on fibroblasts obtained from non-tumor tissue (NFs) and tumor tissue (CAFs) from patients diagnosed with lung adenocarcinoma. Fibroblasts were isolated and cultured for 72 h in the presence or absence of 30 µM THC, 30 µM CBD or a mixture of 10 µM THC and 10 µM CBD, with or without TGFβ in the culture media. Cell number and viability, the distribution of F-actin microfilaments, and the expression of type I collagen, FSP1, and vimentin were evaluated by real-time RT-PCR.

Next, we studied whether cannabinoids modulated the interactions between cancer cells, CAFs and NFs. For this study, we cultured A549 cells with NF- or CAF-conditioned culture medium (CM) treated with or without these drugs, in the presence or absence of TGFβ. A549 cells were cultured with NF or CAF CM and the viability, cell number, and F-actin microfilaments distribution, as well as CDH1, CDH2, and VIM gene expression, were evaluated by real-time RT-PCR. The expression of vimentin and pankeratin was also studied by immunofluorescence. Finally, CAFs and NFs were cultured with A549 cell CM treated or not with TGFβ in the presence or absence of a mixture of cannabinoids. The distribution of F-actin microfilaments and the expression of type I collagen, FSP1 and vimentin were evaluated by immunofluorescence and real-time RT-PCR.

### 2.2. Cell Lines and Cell Isolation

The human lung adenocarcinoma epithelial cell line A549 was purchased from the American Type Culture Collection (ATCC, Rockville, MD, USA). Cells were cultured in A549 cell proliferation medium, composed of RPMI 1640 with stable glutamine (EuroClone, Siziano PV, Italy) supplemented with 5% heat-inactivated fetal bovine serum (HI-FBS, Gibco, Germany), 10 mM HEPES, 1% penicillin–streptomycin (P/S), and 1% amphotericin B (Euroclone, Siziano PV, Italy) in a humidified atmosphere incubator at 37 °C and 5% CO_2_.

Fibroblasts were isolated from lung adenocarcinoma tissues from 3 different diagnosed patients who underwent surgery, as previously reported [22]. The samples were collected at the Hospital La Ribera, from Alzira (Valencia, Spain). This study was conducted in accordance with the ethical standards of the Declaration of Helsinki of the World Medical Association. All the procedures included in this study were approved by the ethics committee of the hospital, and informed consent was obtained from all included patients. To obtain CAFs and NFs, tumor and non-tumor tissues, respectively, were fractionated into small pieces of approximately 3 mm and digested with liberase DH (Sigma-Aldrich, Madrid, Spain) for 90 min under shaking at 37 °C. Samples were filtered through a 70 µm filter (Gibco, Madrid, Spain), washed in sterile PBS (Sigma-Aldrich, Madrid, Spain), and cultured in fibroblast culture medium, composed of high-glucose DMEM culture medium supplemented with 10% HI-FBS, 1% penicillin–streptomycin (P/S), and 1% amphotericin B, in 6-well culture plates (Euroclone, Siziano PV, Italy) in the incubator, until confluence. Adherent cells were expanded and subcultured for a maximum of 6 passages.

### 2.3. Conditioned Medium (CM) Obtaining

A549 cells, NFs and CAFs were grown in their respective culture media to approximately 40% confluency as detailed above. Cells were then cultured in the presence or absence of 5 ng/mL TGFβ, with or without the further addition of 30 µM THC, 30 µM CBD or a mixture of cannabinoids (10 µM THC and 10 µM CBD). After 72 h of culture, media were collected and diluted to 50% with their respective fresh culture medium and stored at 20 °C until later use (conditioned media, CM). Only one freeze cycle was performed.

### 2.4. Cell Viability

Cell viability was determined using the MTS assay (CellTiter 96 Aqueous One Solution Cell Proliferation Assay, Promega, Spain) as previously reported [23]. A549 cells, NFs and CAFs were seeded in 96-well plates at a density of 1 × 10^4^ cells/well. After 24 h of culture, the culture media were removed, and CM, 5 ng/mL TGFβ, or cannabinoids were added, as expressed in the different experiments. Latex CM was used as the positive cytotoxic control and cells cultured with non-conditioned media were also used as a control. After 24 h of culture, the MTS assay was carried out following the manufacturer’s indications using a spectrophotometer at 490 nm.

### 2.5. F-actin Fluorescence Staining and Evaluation of Cell Density

F-actin was evaluated using rhodamine-conjugated phalloidin (Molecular Probes, Thermo Fisher Scientific, Madrid, Spain) as previously described [23]. A549 cells, NFs and CAFs were cultured either in the respective culture medium (treated or not with TGFβ and/or cannabinoids) or with different CM for 72 h. Cells were then washed with PBS pH 7.4 and fixed in a 4% solution of paraformaldehyde in PBS for 10 min at RT. Cells were permeabilized with 0.1% Triton X 100 in PBS for 3–5 min. Then, samples were preincubated with PBS containing 1% BSA for 20 to 30 min to reduce the non-specific background. Each sample was then stained for 20 min with phalloidin (5 µL phalloidin stock solution in methanol diluted in 200 µL PBS). Finally, the samples were washed several times with PBS, and the nuclei were stained with DAPI and analyzed with a DM2500 fluorescence microscope (Leica, Madrid, Spain). Cellular morphology was studied by means of F-actin microfilament distribution, while cell density was estimated by counting the number of nuclei stained with DAPI in 5 fields of each sample at 10x magnification using Image-Pro plus 7 software (Media Cybernetics, USA), and the mean number of nuclei per field was calculated in each of the experimental conditions.

### 2.6. Determination of COL1A1, VIM, FSP1, CDH1, CDH2 and VIM Expression Levels

Total RNA was extracted from cells cultured in their respective media or in different CM for 72 h, using the TRIzol reagent (Thermo Fisher Scientific Inc., Waltham, MA, USA), according to the manufacturer’s instructions. RNA concentration was determined by spectrophotometry using a Nanodrop 2000 spectrophotometer (Fischer Scientific, Madrid, Spain). RNA integrity was assessed by capillary electrophoresis using a Bioanalyzer (Agilent Technologies, Santa Clara, CA, USA). Only extracts with a 260/280 nm ratio > 1.8 and with RIN of ~10 were used for the determination of gene expression levels.

Random hexamers were used to synthesize complementary DNA (cDNA) using TaqMan RT reagents (Applied Biosystems, Foster City, CA, USA), following the manufacturer’s instructions. Gene expression levels were assayed by reverse transcriptase polymerase chain reaction (RT-PCR) using Assays on Demand (Applied Biosystems, Madrid, Spain). The reactions were carried out in a 7900HT Real-Time Thermocycler (Applied Biosystems, Madrid, Spain) [21]. The comparative ΔΔCt method with glyceraldehyde 3 phosphate dehydrogenase (GAPDH) was used as an endogenous control to calculate relative levels of gene expression.

### 2.7. Detection of Type I Collagen, Vimentin, FSP1 and Pankeratin by Immunofluorescence

Protein expression of type I collagen, vimentin, FSP1, and pankeratin in cell cultures was determined using specific antibodies. Cells were cultured in 8-well Millicell glass (Merck, Germany) at a density of 2 × 10^3^ (A549 cells) or 8 × 10^3^ (NFs or CAFs) cells/well. Cells were fixed with 4% paraformaldehyde in PBS pH 7.4 for 10 min at RT. Once washed with PBS, cells were permeabilized with 0.1% Triton X 100 in PBS for 5 min, and after three washes with PBS, they were incubated for 30 min with blocking solution (1% bovine serum albumin and 1.1% Tween 20 in PBS). Cells were then incubated with the appropriate monoclonal primary antibodies diluted in antibody diluent solution. The primary antibodies used were: Cytokeratin Pan-Alexa fluor (1:100 dilution; Invitrogen, USA), Type I Collagen (1:100 dilution; Merck, Germany), Vimentin (2D1; 1:500 dilution; Novus Biologicals, USA) and FSP1 (1:100 dilution; Novus Biologicals, USA). Cells were incubated with the primary antibody overnight at 4 °C and after three washes, they were incubated with the anti-mouse FITC-conjugated secondary antibody (Sigma-Aldrich, Madrid, Spain) diluted 1:200, except cytokeratin, which was carried out by direct immunofluorescence. After the final washes, nuclei were stained with DAPI and samples were analyzed with a Leica DM2500 fluorescence microscope (Leica, Wetzlar, Germany).

### 2.8. Data Presentation and Analysis

Cells from 3 different patients were used for NF and CAF isolation, and all determinations were carried out in triplicate. For microscopy experiments, representative images of 5 fields of each stain are presented. Data related to cell viability, number of nuclei, and gene expression are presented as the mean ± SD. Statistical analysis was carried out using analysis of variance (ANOVA) followed by Tukey’s multiple-comparison test (GraphPad Software Inc., San Diego, CA, USA). Significance was accepted at *p* < 0.05.

## 3. Results

### 3.1. Effect of Cannabinoids on Non-Tumor Fibroblasts’ (NFs) and Cancer-Associated Fibroblasts’ (CAFs) Morphology, Viability and Cell Density

**Figure 1:**
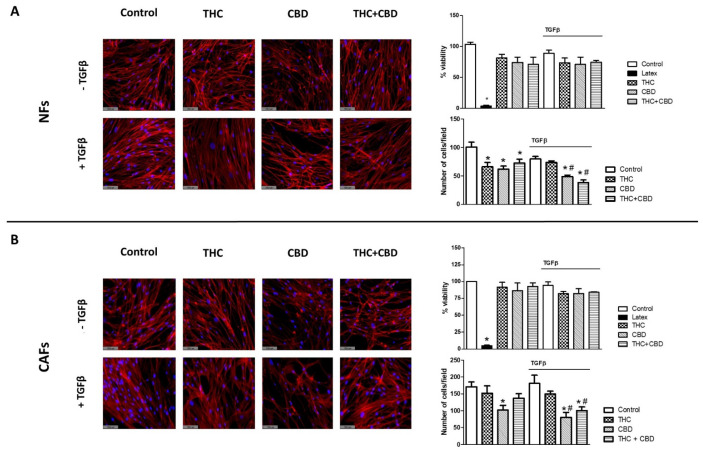
Effect of cannabinoids on cell morphology, viability and cell density in normal fibroblasts (NFs, (**A**)) or cancer-associated fibroblasts (CAFs, (**B**)). Cells were cultured for 72 h in the presence or absence of 5 ng/mL TGFβ and treated or not with 30 µM THC, 30 µM CBD, or an equimolar mixture of 10 µM of THC + CBD. Cellular morphology was studied by fluorescent staining of F-actin microfilaments, using rhodamine-conjugated phalloidin. Representative fluorescence images from to 3 different experiments are shown. The percentage of viability was measured by the MTS assay using latex as a positive control for citotoxicity. Cell density was obtained by counting the number of DAPI-stained cell nuclei in five fields. The mean ± SD of 3 different experiments is represented. A triplicate was analyzed in each of the experiments. * *p* < 0.05 compared to the control group not stimulated with TGFβ. # *p* < 0.05 compared to the TGFβ-stimulated control group. Scale bar is equal to 100.4 µm.

Our first objective in this study was to evaluate whether THC or CBD, alone or in combination, had any effect on the morphology, viability and cell density of stromal cells obtained from patients diagnosed with adenocarcinoma. CAFs were isolated from tumor tissue from three different patients and NFs from non-tumor tissue from the same patients, and the latter were used as controls. Cells were treated with THC, CBD, or THC + CBD, in the presence or absence of TGFβ. Morphology, viability and cell density were evaluated. The results obtained are summarized in Figure 1.

Regarding morphology, both the NFs and CAFs of the control group presented the elongated shape characteristic of fibroblasts, with the presence of a well-organized cytoskeleton with abundant F-actin microfilaments throughout the cytoplasm. Apparently, none of the cannabinoid treatments significantly altered this morphology, while, in general, the cells treated with TGFβ presented a slightly more developed cytoplasm. However, these differences were minimal, and could not be quantified (Figure 1A,B). In the NFs, no significant differences were observed in relation to cellular toxicity with respect to the different drugs or the presence or absence of TGFβ. Regarding cell density, both THC and CBD alone or in combination significantly decreased the number of NFs compared to controls (panel A, *p* < 0.05). The most evident effect was observed in the groups treated with CBD in the presence of TGFβ, with a significant reduction that was at its maximum when CBD was administered in combination with THC. Similar effects were observed in CAFs (panel B), with the greatest decrease in cellularity in the groups treated with 30 µM CBD, both in the presence and absence of TGFβ, as could be observed both in the fluorescence images and in the cell counts performed.

### 3.2. Effect of Cannabinoids on NF and CAF Gene Expression Levels of COL1A1, FSP1 and VIM

**Figure 2:**
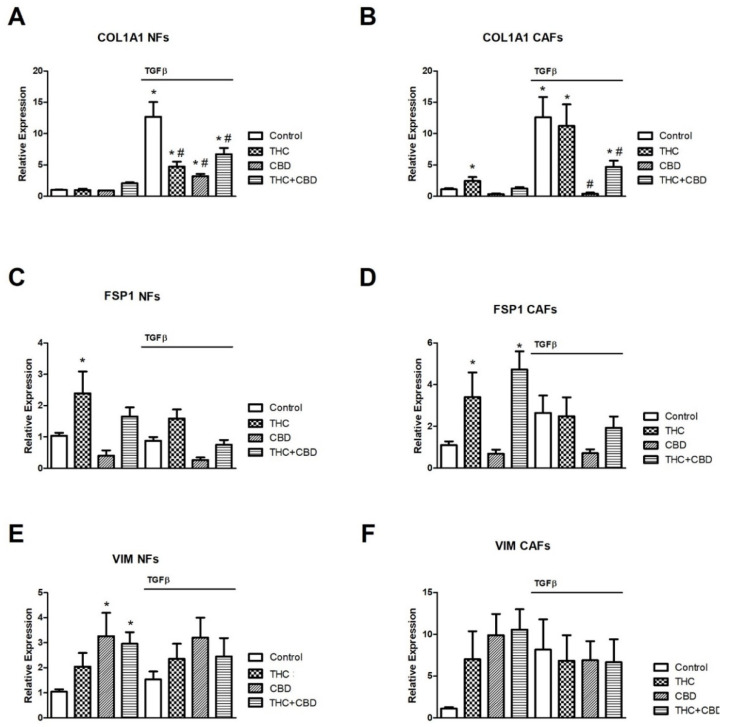
Effect of cannabinoid agonists on fibroblast-related gene expression. NFs (**A**,**C**,**E**) and CAFs (**B**,**D**,**F**) were cultured for 72 h in the presence or absence of 5 ng/mL TGFβ and treated or not with 30 µM THC, 30 µM CBD, or an equimolar mixture of 10 µM THC + CBD. The relative gene expression of COL1A1, FSP1 and VIM was studied by real-time RT-PCR using GAPDH as housekeeping. Average fold change ± SD of 3 different experiments is represented. A triplicate was analyzed in each of the experiments. * *p* < 0.05 compared to the control group not stimulated with TGFβ. # *p* < 0.05 compared to the TGFβ-stimulated control group.

Next, we studied whether cannabinoid treatment had any effect on the expression of genes characteristic of CAF differentiation. For this study, CAFs from three patients and their respective NFs were cultured and treated with THC and/or CBD in the presence or absence of TGFβ, as described above. After 72 h of culture, total RNA was extracted and the relative expression of COL1A1, FSP1 and VIM, all in relation to GAPDH expression, was studied as described in the methods section. The results obtained are summarized in Figure 2.

The exposure of CAFs and NFs to TGFβ induced, as expected, a significant increase in COL1A1 expression in the control groups (Figure 2A,B). In the case of CAFs, THC did not generate any inhibitory effect, while CBD completely abolished the increase induced by TGFβ, both alone and in combination with THC, although substantially less inhibition was observed in this case (panel B). In the case of NFs, a different behavior was observed. Both THC and CBD alone or in combination significantly inhibited TGFβ-induced COL1A1 expression, although once again, the greatest inhibitory effect was observed in cultures treated with 30 µM CBD (panel A). We did not observe significant effects in relation to the expression of the COL1A1 gene in cultures not exposed to TGFβ, both in NFs and in CAFs (panels A,B), except for a small increase in CAFs exposed to THC.

Regarding FSP1 expression, we detected significant increases in CAFs treated with THC (alone or in combination with CBD) in the absence of TGFβ (panel D), and FSP1 was also significantly increased in NFs cultured with THC alone in the absence of TGFβ (panel C). In addition, we did not find significant changes in the expression of VIM in the studies carried out with CAFs (panel F), while in those with NFs we could only observe a significant increase in the groups treated with CBD alone or in combination with THC, in the absence of TGFβ (panel E).

### 3.3. Stromal Cell CM Induces Changes in A549 Cells’ Morphology and Density and Efficacy of Treatment with THC and/or CBD

**Figure 3:**
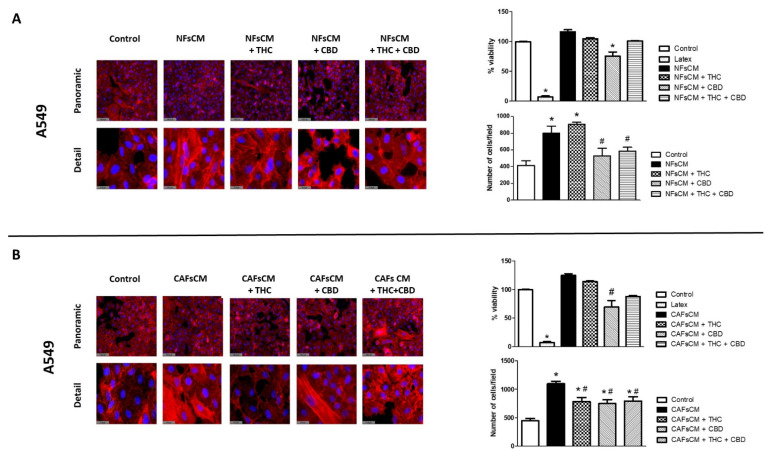
Effect of cannabinoid agonists on A549 cell morphology, viability, and cell density changes induced by stromal-cell-conditioned medium. Culture medium was conditioned with NFs (NFsCM) or CAFs (CAFsCM) treated or not with 30 µM THC, 30 µM CBD, or an equimolar mixture of 10 µM THC + CBD. A549 cells were cultured with NFsCM (**A**) or CAFsCM (**B**) for 72 h. Cellular morphology was studied by the fluorescent staining of F-actin microfilaments, using rhodamine-conjugated phalloidin. Representative fluorescence images corresponding to 3 different experiments are shown. The percentage of viability was measured by the MTS assay using latex as a positive control for cytotoxicity. Cell density was obtained by counting the number of DAPI-stained cell nuclei in five fields. The mean ± SD of 3 different experiments is represented. A triplicate was analyzed in each of the experiments. * *p* < 0.05 compared to the control group (white bars). # *p* < 0.05 compared to the NFsCM or CAFsCM in the absence of cannabinoid agonists (black bars). Scale bar is equal to 100.4 (panoramic images) or 31.5 (detail images) µm.

We next studied whether stromal cells isolated from adenocarcinoma patients had any effect on morphology, viability, or cell density in A549 cells. For this study, we obtained NF or CAF CM treated without TFGβ, in the presence or absence of THC and/or CBD, as described in the methods section. Then, we cultured A549 cells with those CMs, using non-conditioned media as the control. The results are summarized in Figure 3. A549 cells cultured in non-conditioned media showed the characteristic epithelial morphology, with polygonal cells intimately attached to each other (Figure 3A,B). When A549 cells were exposed to NF or CAF CM, a change in cell morphology was induced towards the fibroblastic phenotype, with the appearance of more elongated, spindle-shaped cells, with thicker F-actin microfilaments and wider intercellular spaces. Cells cultured with NF or CAF CM treated with cannabinoids did not show these morphological changes but maintained a more epithelial phenotype, similar to that of the control group. These morphological changes were more noticeable in those groups treated with CBD, alone or in combination with THC. Regarding cell viability, no significant changes were observed, except in those cells cultured in NF and CAF CM treated with 30 µM CBD alone, which showed small but significant decreases. On the other hand, cell density was significantly higher in A540 cells cultured with NF and CAF CM, in which both THC and CBD, alone or in combination, significantly inhibited these increases, except when exposed to NF CM treated with 30 µM THC alone.

### 3.4. Changes in EMT Gene Expression in A549 Cells Cultured with Stromal Cell CM and Effect of THC and CBD

**Figure 4:**
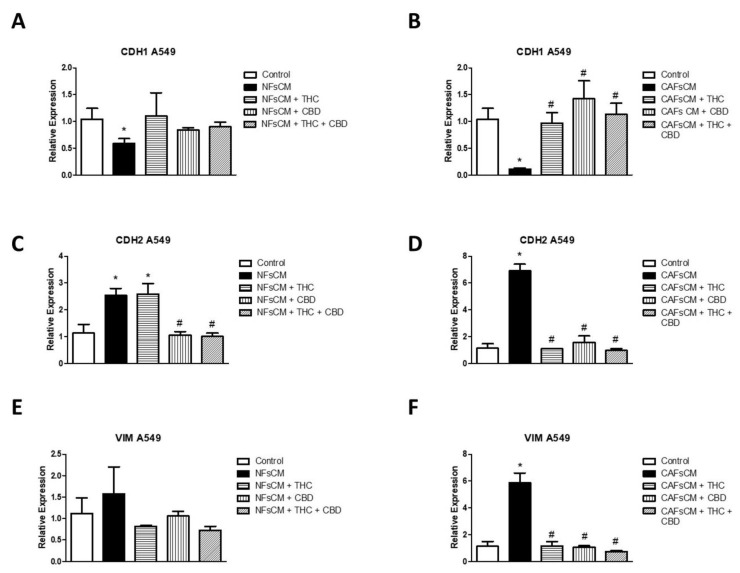
Effect of cannabinoid agonists on A549 cell gene expression of EMT-related gene changes induced by stromal-cell-conditioned medium. The culture medium was conditioned with NFs (NFsCM) or CAFs (CAFsCM) treated or not with 30 µM THC, 30 µM CBD, or an equimolar mixture of 10 µM THC + CBD. A549 cells were cultured with NFsCM (**A**,**C**,**E**) or CAFsCM (**B**,**D**,**F**) for 72 h. The relative gene expression of CDH1, CDH2 and VIM was studied by real-time RT-PCR using the GAPDH as housekeeping. Average fold change ± SD of 3 different experiments is represented. A triplicate was analyzed in each of the experiments. * *p* < 0.05 compared to the control group (white bars). # *p* < 0.05 compared to NFsCM or CAFsCM in the absence of cannabinoid agonists (black bars).

Next, we studied whether the changes observed at the phenotypic level correlated with changes in the expression of three genes related to EMT (Figure 4). We cultured A549 cells with NF or CAF CM treated with or without THC and/or CBD. When comparing A549 cells cultured with NF CM with the control group, we observed a significant inhibition in CDH1 relative gene expression (Figure 4A), and an increase in CDH2 expression (panel C), whereas the increase observed in VIM expression was no significant (panel E). Greater changes were observed in the experimental groups cultured with CAF CM, with a significant decrease for CDH1 and an increase in the relative expression of CDH2 and VIM (panels B,D,F). Regarding the treatment with cannabinoid agonists, we observed a different behavior in relation to NF or CAF CM. None of the treatments with cannabinoid agonists managed to reverse the inhibitory effect of CDH1 expression in the groups cultured with NF CM, while all the formulations used with CAF CM managed to reverse this effect (panels A,B). Regarding the expression of CDH2, THC failed to inhibit the increase observed in A549 cells cultured with NF CM, while it drastically reversed the increase observed in those cultured with CAF CM, treated with THC and/or CBD (panels C,D). Regarding the relative expression of VIM, no significant variations were observed in cells cultured with NF CM, unlike those cultured with CAF CM, where the significant increase observed in VIM expression was significantly reversed in all groups treated with THC and/or CBD (panels E,F).

### 3.5. Effect of Stromal Cell CM and Cannabinoid Agonists on A549 Cell Expression and Distribution of Vimentin and Pankeratin Proteins

**Figure 5:**
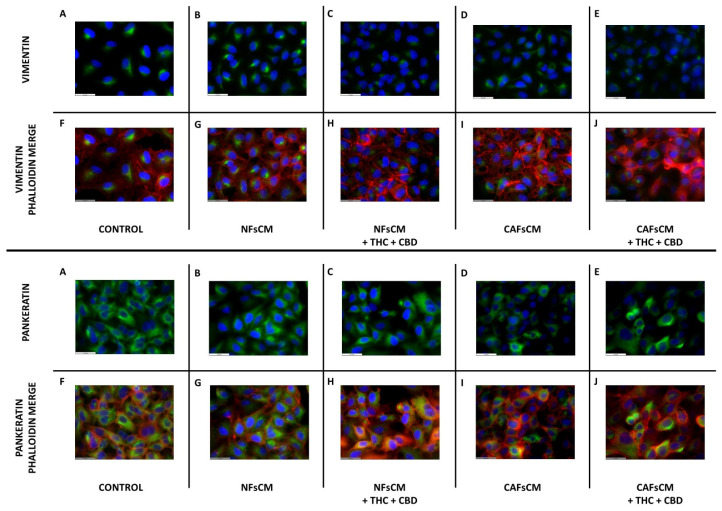
Effect of cannabinoid agonists on A459 cell cytoskeletal changes induced by stromal-cell-conditioned medium. The culture medium was conditioned with NFs (NFsCM) or CAFs (CAFsCM) treated or not with an equimolar mixture of 10 µM THC + CBD. A549 cells were cultured with non-conditioned culture medium (**A**,**F**), NFsCM (**B**,**G**), NFsCM + THC + CBD (**C**,**H**), CAFsCM (**D**,**I**) or CAFsCM + THC + CBD (**E**,**J**) for 72 h. Vimentin and cytokeratin filaments were studied by immunofluorescence. F-actin was evaluated using rhodamine-conjugated phalloidin. Representative fluorescence images corresponding to 3 different experiments are shown. A triplicate was analyzed in each of the experiments. Scale bar is equal to 31.5 µm.

Then, we decided to study whether there was any change in the expression and distribution of vimentin and pankeratin filaments in A549 cells cultured with NF or CAFCM. For these experiments, and given the effects observed in the gene expression experiments, we only evaluated the effect of the cannabinoid mixture (THC + CBD, 10 µM each). Representative results are shown in Figure 5. Regarding vimentin, basal expression was detected in the control group and in the groups cultured with NF or CAF CM without cannabinoids treatment (Figure 5A,B,D). Cannabinoid treatment slightly decreased vimentin expression, but no changes were observed regarding its intracellular distribution in either NF or CAF CM (panels C,E). Regarding pankeratin, abundant expression was observed in control cells (panel A), and only a minimal decrease in its expression was observed in A549 cells cultured with CAF CM without cannabinoid treatment (panel D), which were of small magnitude and reversed by the mixture of cannabinoids used (panel E).

### 3.6. A549 Cell CM Stimulates CAF and NF Expression of Type I Collagen, Vimentin and FSP1 and Effect of Cannabinoids

**Figure 6:**
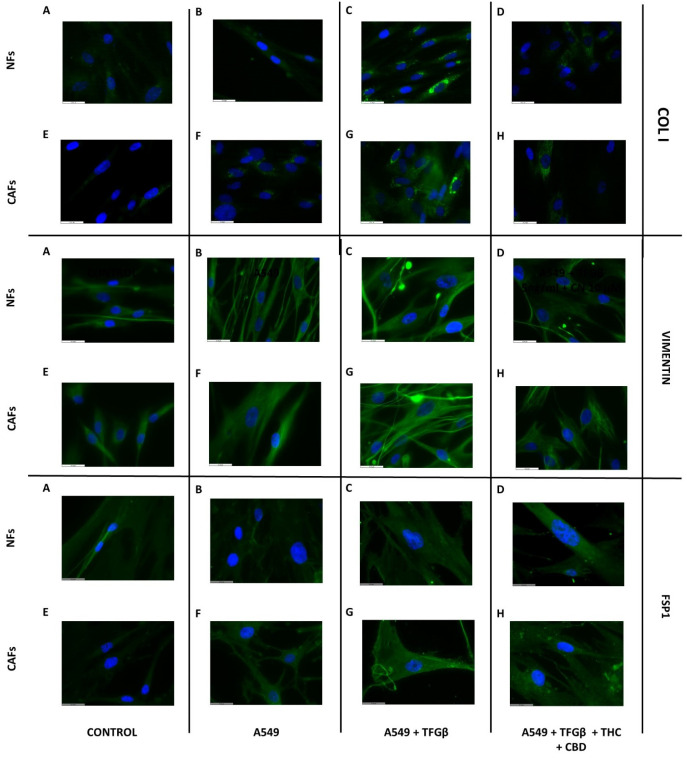
Effect of cannabinoid agonists on stromal cell cytoskeletal changes induced by A549-cell-conditioned medium. NFs or CAFs were cultured for 72 h with non-conditioned culture medium (**A**,**E**), A549-cell-conditioned medium (A549CM, (**B**,**F**)), A549CM + 5 ng/mL TGFβ (**C**,**G**) or A549CM + 5 ng/mL TGFβ + an equimolar mixture of 10 µM THC + CBD (**D**,**H**). Type I collagen, vimentin and FSP1 were studied by immunofluorescence. Representative fluorescence images corresponding to 3 different experiments are shown. A triplicate was analyzed in each of the experiments. Scale bar is equal to 31.5 µm.

Next, we wanted to evaluate whether A549 cancer cells could influence the behavior of NFs and CAFs. Thus, we obtained CM from A549 cells treated or not with 5 ng/mL TGFβ in the presence or absence of the cannabinoid mixture (THC + CBD 10 µM each). Representative results are shown in Figure 6. No effect on type I collagen protein expression was observed in cells cultured with A549 cell CM without TGFβ treatment (Figure 6B,F). However, a marked increase in both NFs and CAFs cultured with A549 cell CM treated with TGFβ (panels C,G) was observed, which was inhibited when exposed to the cannabinoid mixture (THC + CBD 10 µM each; panels D,H). A similar trend was observed for vimentin expression, which, like type I collagen expression, showed no changes in cells cultured with A549 CM with respect to the controls (panels B,F), while an increase was observed when cultured with TGFβ (panels C,G), which was inhibited in the presence of cannabinoids, with a greater magnitude in CAFs than in NFs (panels D,H). Regarding FSP1 protein expression, no significant variations were observed in the NFs (panels A–D), while in the CAF cultures we observed a slight increase in the groups cultured with A549 cell CM treated with TGFβ (panel G), but no significant variations were observed in relation to cannabinoid exposure (panel H).

### 3.7. Effects of A549 Cell CM on Gene Expression of COL1A1, VIM and FSP1 in NFs and CAFs and Effects of Cannabinoids

**Figure 7:**
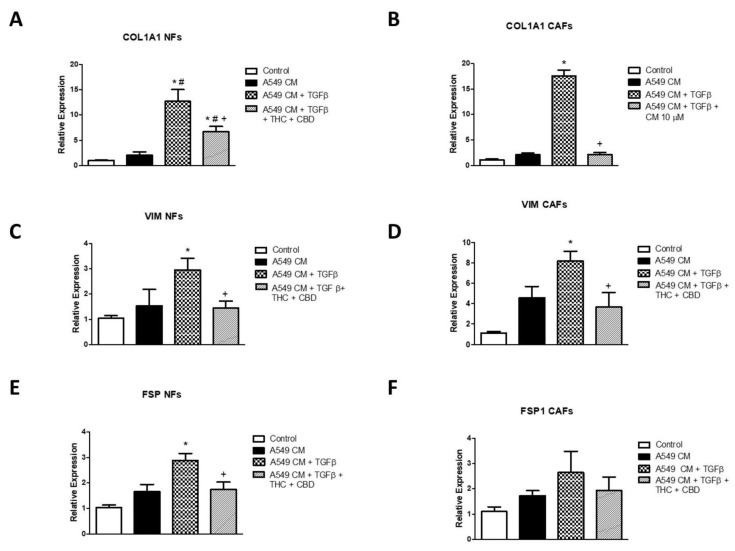
Effect of cannabinoid agonists on stromal cell gene expression of fibroblast-related gene changes induced by A549-cell-conditioned medium (A549CM). The culture medium was conditioned with A549 cells cultured in the presence or absence of 5 ng/mL TGFβ, treated or not with an equimolar mixture of 10 µM THC + CBD. Then, NFs (**A**,**C**,**E**) or CAFs (**B**,**D**,**F**) were cultured with this conditioned medium for 72 h. The relative gene expression of COL1A1, VIM and FSP1 was studied by real-time RT-PCR using the GAPDH as housekeeping. Average fold change ± SD of 3 different experiments is represented. A triplicate was analyzed in each of the experiments. * *p* < 0.05 compared to the control group (white bars). # *p* < 0.05 compared to the NFs or CAFs cultured with A549CM in the absence of TGFβ or cannabinoid agonists (black bars). + *p* < 0.05 compared to the NFs or CAFs cultured with A549CM + TGFβ in the absence of cannabinoid agonists (gridded bars).

Finally, we studied whether the changes observed by immunofluorescence were correlated with changes at the level of gene expression. For this study, we cultured NFs and CAFs with A549 cell CM, including the same experimental groups as in the previous section. We analyzed the expression of type I collagen, vimentin and FSP1 by real-time RT-PCR. The results obtained are summarized in Figure 7. We observed a significant increase in the expression of COL1A1 in cells cultured with A549 cell CM treated with TGFβ. This increase was of greater magnitude in the case of CAFs (Figure 7A,B). In both cases, the cannabinoid mixture significantly reduced this increase, which was more marked in the case of the CAF cultures. A similar profile was observed in the case of the relative expression of VIM, where the induction was also higher in CAFs than in NF cultures. In both cases, cannabinoid agonists reversed this increase (panels C,D). Finally, FSP1 expression showed a significant increase in NFs cultured with A549 cell CM treated with TGFβ, which was also inhibited by cannabinoid treatment (Figure 7E). In the case of CAFs, although a similar trend was observed, the changes did not reach statistical significance (Figure 7F). 

## 4. Discussion

NSCLC is the leading cause of cancer-related mortality in the United States today [24,25]. There are different factors involved in the great malignancy of NSCLC, including its great metastatic capability and ability to generate resistance against currently used chemotherapeutic agents [26,27]. This situation has led numerous researchers, including our group, to investigate new drugs, in which cannabinoid agonists are included, that can complement existing treatments to alleviate the effects of this terrible disease [28].

As we have already mentioned, cannabinoid agonists, in addition to their analgesic, antianorexic and antiemetic effects, have shown their utility in different in vitro and in vivo models of cancer, including lung cancer [29]. The combined use of THC and powerful CBD has shown the potential to enhance these effects, also reducing the harmful effects of THC at the level of the central nervous system. In fact, there is an FDA-approved drug called Sativex©, which is made up of an equimolar combination of these molecules and is indicated for the treatment of pain and other disorders, including multiple sclerosis [30]. We previously reported the effect of THC and CBD alone or in combination in an in vitro model carried out on three different cancer cell lines, which showed that CBD potentiated the effects of THC in terms of the inhibition of EMT proliferation and development in these three cell lines [20]. However, the use of only cell lines for the study of antitumor drugs, although it has been useful, has serious deficiencies in relation to the impossibility to study the effects of such drugs on the complexity of the tumor microenvironment (TME), which includes not only characteristic stromal proteins, but also endothelial, mesenchymal, and inflammatory cells, along with CAFs [31]. CAFs are one of the key elements in the control of the TME. CAFs derive from different cell types, including connective tissue-resident fibroblasts, mesenchymal cells, and even cancerous epithelial cells, which undergo extreme epithelial-to-mesenchymal transition (EMT) [32]. CAFs control the composition of the tumor stroma and induce, among other things, EMT and metastasis in lung cancer, which makes them an attractive pharmacological target in the treatment of this pathology [30,32]. In the present study, we show that treatment with THC and/or CBD decreases cell density and the expression of type I collagen and FSP1 in CAFs isolated from the tumors of patients diagnosed with lung adenocarcinoma. These effects are different from those observed in normal fibroblasts (NFs), isolated from the healthy peripheral tissue of the same patients, as demonstrated by the results related to the expression of vimentin, which reinforces the differential behavior between both cell types. Furthermore, we observed an additive effect of the combined used of THC and CDB on these parameters, which allowed us to obtain similar effects with lower doses of THC, thus reducing the psychotoxic effects of this molecule. Hamad et al. reported that CBD induced the cell death of human lung cancer stem cells [33], but, to our knowledge, this is the first study reporting the effects of the combined use of THC and CBD on CAFs and NFs isolated from the same patients with lung cancer. In this work we used doses of 30 µM for THC and CBD when they were administered alone and 10 µM in combination. These doses correspond to the published IC50 doses for A549 cells [20].

Although these results are interesting in themselves, they must be considered in the context of the TME. Therefore, it is necessary to evaluate the interactions that occur between the cellular and non-cellular components of the TME, which is currently our main line of research. We recognize the complexity of the TME and do not intend to solve it as a whole. That is why we designed a simple system that consists of stimulating CAFs or NFs with A549-cell-conditioned culture medium treated or not with THC and/or CBD. On the other hand, we also studied the effect of stimulating A549 cells with CAF- or NF-conditioned media treated with or without the same drugs. Furthermore, we introduced TGFβ in our design, since it is a key element in the regulation of the TME [34]. We used short TGF stimulation times to determine the effect of THC and CBD as blockers of the early stages of EMT in A549 cells. Conditioned culture media (CM) have been used by other researchers, and although they are a model that has some deficiencies, they allow establishing the existence of relationships between different cells in a simple way [35]. One of the limitations of this study resides precisely in the impossibility of characterizing the composition of these culture media, although we think that it is a good starting point to establish future models in which we could characterize these elements.

The stimulation of A549 cells with NF or CAF CM induced an increase in cell number, higher in the case of CAFs, which was partially inhibited by the cannabinoid pretreatment of stromal cells. This inhibition was more effective in A549 cells cultured with CAF CM, highlighting the differences found in relation to the origin of the stromal cells. A very interesting aspect is the induction of a gene expression profile compatible with EMT, in relation to the expression of CDH1, CDH2 and VIM, in A549 cells cultured with these conditioned media. Again, these changes were more pronounced with CAF CM, which could be due to a greater release of factors such as TGFβ, although this aspect should be explored in more detail [36]. Once again, treatment with THC and CBD, alone or in combination, inhibited these changes, reinforcing the beneficial use of both drugs in combination, since we observed similar effects but using a dose three times lower of these compounds. It would be interesting to study this effect in more representative models of the TME, which is one of our objectives for the future.

The immunofluorescence study of the vimentin and pankeratin filaments showed a slight modification in their distribution, which was inhibited by the combined use of THC + CBD. In relation to vimentin, it is noteworthy that A549 cells have a certain mesenchymal phenotype, which explains their basal expression. The slight decrease in pankeratin is compatible with cellular transformation from an epithelial phenotype towards a more mesenchymal one, as pointed out by other authors [37].

The culture of NFs and CAFs with A549 cell CM also generated changes at the morphological and gene expression levels. In this case, we did not observe changes in A549 cells cultured with these conditioned media in the absence of TGFβ. It might be thought that this effect would be generated by the same TGFβ used for the stimulation of A549, but it is important to point out that we used a very low dose, only 5 ng/mL, which we later diluted by half, so that the dose of exogenous TGFβ added to NFs and CAFs was 2.5 ng/mL at most. We carried out further experiments using this dose as a direct stimulus in the fibroblasts, but we did not observe any effect. These data are not included in the manuscript in order to simplify the figures. The stimulation of NFs and CAFs with these media induced an increase in the expression of type I collagen, vimentin and FSP1, which demonstrates the acquisition of a more secretory phenotype, characteristic of the tumor stroma. In relation to FSP1, a greater increase in its relative expression was observed in the case of NFs, probably because the basal expression of this gene is higher in CAFs than in NFs [38].

In summary, the results shown here strengthen the possible beneficial effect of cannabinoid agonists, not only on the tumor cells themselves, but also on CAFs, key elements of the TME. Although the experimental model used is rudimentary, these results reinforce the need to study the effect of these drugs in more complex models that could represent the complexity of the TME in a more realistic way.

## Data Availability

Not applicable.
